# High-Frequency Stimulation of the Subthalamic Nucleus Counteracts Cortical Expression of Major Histocompatibility Complex Genes in a Rat Model of Parkinson’s Disease

**DOI:** 10.1371/journal.pone.0091663

**Published:** 2014-03-12

**Authors:** Benjamin Grieb, Gerhard Engler, Andrew Sharott, Constantin von Nicolai, Thomas Streichert, Ismini Papageorgiou, Alexander Schulte, Manfred Westphal, Katrin Lamszus, Andreas K. Engel, Christian K. E. Moll, Wolfgang Hamel

**Affiliations:** 1 Department of Neurophysiology and Pathophysiology, University Medical Center Hamburg-Eppendorf, Hamburg, Germany; 2 Department of Clinical Chemistry, University Medical Center Hamburg-Eppendorf, Hamburg, Germany; 3 Division of General Neurophysiology, Institute of Physiology and Pathophysiology, University of Heidelberg, Heidelberg, Germany; 4 Department of Neurosurgery, University Medical Center Hamburg-Eppendorf, Hamburg, Germany; 5 Department of General Psychiatry, Center for Psychosocial Medicine, University of Heidelberg, Heidelberg, Germany; 6 Centre for Integrative Neuroscience, University of Tübingen, Tübingen, Germany; University of Naples Federico II, Italy

## Abstract

High-frequency stimulation of the subthalamic nucleus (STN-HFS) is widely used as therapeutic intervention in patients suffering from advanced Parkinson’s disease. STN-HFS exerts a powerful modulatory effect on cortical motor control by orthodromic modulation of basal ganglia outflow and via antidromic activation of corticofugal fibers. However, STN-HFS-induced changes of the sensorimotor cortex are hitherto unexplored. To address this question at a genomic level, we performed mRNA expression analyses using Affymetrix microarray gene chips and real-time RT-PCR in sensorimotor cortex of parkinsonian and control rats following STN-HFS. Experimental parkinsonism was induced in Brown Norway rats by bilateral nigral injections of 6-hydroxydopamine and was assessed histologically, behaviorally, and electrophysiologically. We applied prolonged (23h) unilateral STN-HFS in awake and freely moving animals, with the non-stimulated hemisphere serving as an internal control for gene expression analyses. Gene enrichment analysis revealed strongest regulation in major histocompatibility complex (MHC) related genes. STN-HFS led to a cortical downregulation of several MHC class II (RT1-Da, Db1, Ba, and Cd74) and MHC class I (RT1CE) encoding genes. The same set of genes showed increased expression levels in a comparison addressing the effect of 6-hydroxydopamine lesioning. Hence, our data suggest the possible association of altered microglial activity and synaptic transmission by STN-HFS within the sensorimotor cortex of 6-hydroxydopamine treated rats.

## Introduction

In Parkinsońs disease (PD), nigrostriatal dopamine depletion is the source of severe disturbances within skeletomotor loops that tightly link cortex, basal ganglia and thalamus [1], [2]. Pathological activity originating anywhere in these loops disrupts physiological processing at the level of the sensorimotor cortex and aberrant or missing corticofugal motor commands lead to the emergence of cardinal motor signs in PD, such as akinesia, rigidity and resting tremor. During the last two decades, chronic high-frequency stimulation (HFS, also termed deep brain stimulation, DBS) of the subthalamic nucleus (STN) has emerged as a safe and effective treatment option for medically refractory PD patients [3], [4]. Although the efficacy of STN-HFS in treating PD has been conclusively shown, there are significant differences in the latencies for the amelioration of the abovementioned motor symptoms. While an improvement of rigidity typically occurs immediately and remains stable throughout continuous STN-HFS over several months, an amelioration of resting tremor, bradykinesia and off-drug dystonia is often observed with different latencies ranging from seconds to weeks of continuous HFS [5]. Moreover, return to baseline symptom severity is variable after HFS-offset [6]. Such latency differences and carry-over effects suggest that in addition to immediate electrophysiological or neurochemical modulation of neuronal activity, HFS leads to adaptive and plastic changes on a longer time-scale. This view is supported by experimental evidence that STN-HFS induces synaptic plasticity in the rat STN [7] and mediates neuroprotection on substantia nigra dopaminergic neurons in a model of neurotoxin-induced degeneration [8], [9].

In order to understand the mechanism of action of STN-HFS, a multitude of studies have focused on its immediate consequences on neural activity in different interconnected structures. While some studies favor an involvement of cortico-basal ganglia loops [10], [11], a different view highlights the importance of a retrograde cortical activation via the “hyperdirect” pathway containing projections from frontal cortical areas to the STN [12]–[14]. In fact, a recent study demonstrated that sensorimotor cortex is antidromically activated by STN-HFS [15]. This activation may be of critical importance for amelioration of motor deficits induced by nigrostriatal dopamine depletion [16], [17]. Consistent with this, scalp potentials with short latency have been recorded from surgically treated patients during both low [18]–[21] and high frequency STN stimulation [22].

Whatever the mechanism, virtually all hypotheses converge upon a close involvement of the cerebral cortex in the mediation of STN-HFS effects, as the motor cortex is the origin of the final common pathway for all motor symptoms. While it is clear that STN-HFS immediately alters the discharge characteristics in remote brain areas such as sensorimotor cortex, the long-term adaptive impact of such changes is not clear, and studies investigating the impact of therapeutic STN-HFS on remote brain areas at the molecular level are sparse. We have previously shown that STN-HFS increases the expression of immediate early genes in various nuclei of the basal ganglia and the sensorimotor cortex of naïve rats [23], indicating enhanced neuronal activity in these areas [24]. A recent genetic screening study applied chronic HFS to the thalamus of naïve rats with a focus on changes in hippocampus, striatum and motor cortex [25]. Another study investigated the effects of STN-HFS on gene expression within the basal ganglia of anesthetized hemiparkinsonian rats [26]. However, STN-HFS-associated gene expression changes at the cortical level of awake, parkinsonian animals are hitherto unexplored. Therefore, the present study was specifically designed to elaborate the impact of STN-HFS on sensorimotor cortex at the molecular level in a bilateral PD rat model.

To this end, we employed prolonged unilateral STN-HFS (23 h) in awake and unrestrained rats rendered parkinsonian by means of bilateral 6-hydroxydopamine (6-OHDA) injections into the substantia nigra pars compacta (SNc). Our animal model was characterized in detail by a multi-modal approach consisting of histology, behavioral analysis of locomotion and both intraoperative and postoperative electrophysiology. Affymetrix microarrays and real time RT-PCR analysis were utilized to identify genes regulated by STN-HFS in tissue samples taken from the rat sensorimotor cortex. Western blotting was utilized to investigate protein levels of selected candidate genes.

## Materials and Methods

### Animal model and experimental design

**Figure 1:**
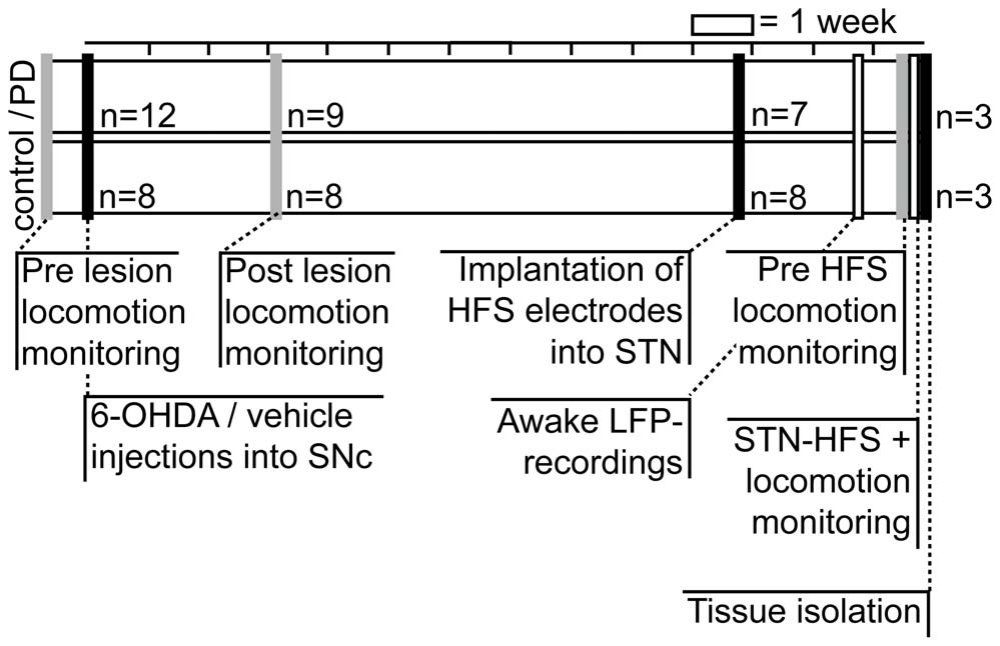
Flowchart of the experimental design. The sequence of experimental steps (i.e., 6-OHDA or vehicle injections, pre- and post-operative locomotion monitoring, STN-HFS electrode implantation, awake LFP recordings, pre-HFS locomotion monitoring, awake STN-HFS under locomotion monitoring, tissue isolation), number of included animals and time between steps is given in a flowchart. 6-OHDA, 6-hydroxydopamine; STN, subthalamic nucleus, HFS, high-frequency stimulation; LFP, local field potential.

Experiments were approved by the local government authorities of Hamburg (Germany; Behörde für Soziales, Familie, Gesundheit und Verbraucherschutz; Fachabteilung für Lebensmittelsicherheit und Veterinärwesen) and carried out in accordance with the European Council Directive 86/609/EEC. All surgeries were performed under ketamine/xylazine anesthesia and all efforts were made to minimize suffering. We randomly assigned 20 male Brown Norway rats (Rattus norvegicus; Charles River Laboratories, Sulzfeld, Germany) to two different experimental groups (see Figure 1): A PD group with 12 rats receiving bilateral 6-OHDA injections into the SNc for induction of experimental parkinsonism [27], [28] and a control group with eight rats receiving bilateral vehicle injections. High mortality rates due to abulia and weight loss are known from bilateral 6-OHDA rats [29]. Therefore, all rats were monitored on a daily basis and offered a soft and moist rat chow and 10%-glucose solution in addition to standard chow and water ad libitum. To sustain aphagic and adipsic PD rats in the most critical post-operative phase, we administered a liquid and high caloric nutrition for rodents (Altromin, Lage, Germany) by manual needle feeding. Animals received s.c.-injections of metamizole (100 mg/kg, Medistar, Holzwickede, Germany) for postoperative analgesia following surgery and on behavioral signs of pain distress (e.g., vocalization upon gentle palpation or self-mutilation such as licking, biting, scratching or other form of harming body parts). Daily inspections took place until three weeks after each surgery. Spontaneous locomotor activity was assessed prior to and three weeks after 6-OHDA lesions. In a second surgery eleven weeks after 6-OHDA injections, we implanted 15 rats (n = 7 PD and n = 8 controls) with bilateral stimulation electrodes into both STNs. Recordings of cortical and subthalamic local field potentials (LFP) took place 12–13 weeks after 6-OHDA lesions. Gene expression profiling following 23 hours of STN-HFS was subsequently carried out 14 weeks after 6-OHDA lesions in a subset of six animals (n = 3 PD, n = 3 controls). Drop outs included perioperative death (n = 2 controls), loss of implant (n = 3 PD) and euthanasia by cervical dislocation under deep anesthesia (i.p.-injection of ketamine 100 mg/kg, xylazine 6 mg/kg) following loss of >20% body weight without stabilization of weight loss within 14 days after surgery (n = 3 PD), development of severe dystonia (n = 1 PD), neoplasia (n = 1 control) or broken stimulation leads (n = 1 PD, n = 1 control).

### Bilateral 6-hydroxydopamine lesions

Stereotactic surgery was performed in rats (preoperative weight: 384±32 g, mean±SD) under general anesthesia. 30 minutes prior to 6-OHDA or vehicle injections, rats received a bolus i.p.-injection of desipramine (25 mg/kg, Sigma-Aldrich, Munich, Germany) to minimize uptake of 6-OHDA in noradrenergic midbrain neurons [27]. Anesthesia was introduced with isoflurane (Baxter Germany GmbH, Unterschleiβheim, Germany) and maintained with i.p.-injections of ketamine (65 mg/kg, Dr. E. Gräub AG, Bern, Switzerland) and xylazine (3 mg/kg, Bayer Health Care, Leverkusen, Germany). An initial bolus of atropine (0.25 mg/kg, B. Braun Melsungen AG, Melsungen, Germany) was administered for cardiopulmonary protection. During surgery we monitored body temperature with a rectal probe and prevented hypothermia with an adjustable heating pad (FST, Heidelberg, Germany). In addition, the eyes were covered with dexpanthenol cream to prevent exsiccation. Animals were mounted in a stereotactic frame (David Kopf Instruments, Tujunga, USA) to target the SNc at +4 mm AP and ±2.2 mm ML using the interaural line as reference [30]. After placement of burr holes, a Hamilton microliter-syringe (FST) was lowered to the target depth at –8 mm relative to the dura. 5 μl neurotoxin (3 μg/μl 6-OHDA in 0.2% ascorbic acid solution, stored on ice; Sigma-Aldrich) or vehicle (aqua injectabilia, 0.2% ascorbic acid solution; Sigma-Aldrich) was slowly infused at a rate of 0.5 μl/min and the syringe was left in place for two minutes to allow for complete absorption of the toxin. The burr hole was closed with bone wax and the procedure was repeated in the contralateral hemisphere.

### Implantation of STN electrodes

**Figure 2:**
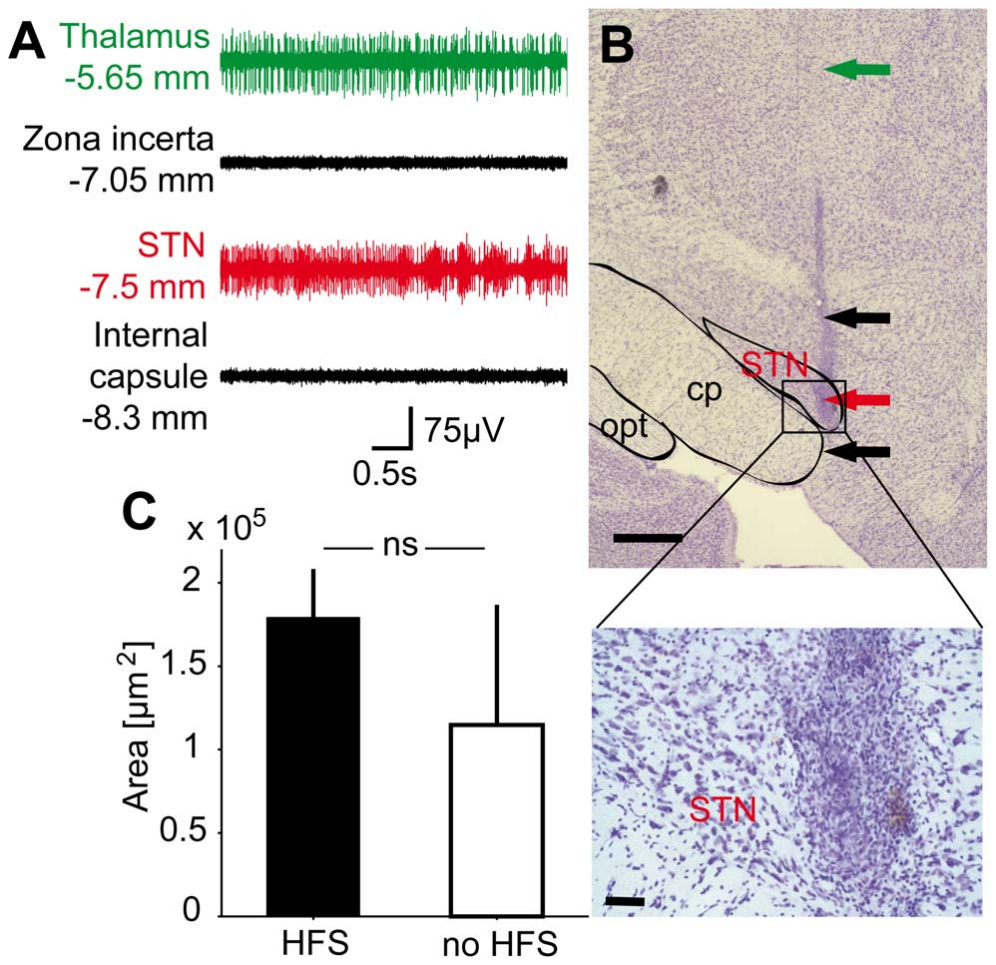
Electrode placement and tissue reaction. Representative traces (A) of microelectrode guided targeting of the STN under ketamine/xylazine anesthesia. Note that the STN trace is characterized by elevated background activity, bursts and irregular high-frequency firing. A representative electrode trajectory (B) targeting the STN (cresyl-violet staining, arrowheads indicate trajectory, scale bar 500 μm). The STN, cerebral peduncle (cp) and optic tract (opt) are circumscribed with black surrounding lines. Photomicrographs of the STN at 20x- and 40x-magnification (scale bars 100 μm and 50 μm, respectively) reveal mild reactive tissue infiltration with mononuclear cells at the electrode tip. Bars (C) indicate the area (mean±SD) infiltrated by mononuclear cells at the tip of stimulated (HFS, black bar) and non-stimulated electrodes (no HFS, white bar; n = 6 in each group). STN, subthalamic nucleus; HFS, high-frequency stimulation; cp, cerebral peduncle; opt, optic tract.

STN electrodes were implanted in a second surgery 54±11 days after SNc surgery in 15 rats (n = 7 PD; n = 8 controls; see Figure 1). The experimental design incorporated a long time window between the first and second surgery to allow PD rats to fully recover from lesioning. Fine needle electromyography electrodes (Technomed, Beek, The Netherlands) were customized for prolonged application of STN-HFS [31]. To minimize risk of electrical tissue damage during HFS, electrodes consisted of a gold-plated stainless steel cannula (350 μm shaft diameter, isolated with coating varnish, Beck Electrical Insulation, Hamburg, Germany) with an isolated inlay of platinum-iridium wire (50 μm tip diameter, cathode). Furthermore, each electrode tip was checked microscopically to ensure that the platinum-iridium inlay was completely embedded inside the cannula forming a clean cut surface without sharp edges, thereby avoiding the occurrence of high current densities at the cathode [32]. After exposure of the skull, we drilled two burr holes (1.8 mm diameter; +5 mm AP, ±2.2 mm ML relative to the interaural line) for microelectrode navigation using two tungsten microelectrodes (FHC, Bowdoin, USA; 200–800 kΩ impedance; 0.5 mm spacing) mounted on a manual electrode microdrive (Alpha Omega, Nazareth, Israel). Signals from each electrode were pre-amplified, amplified and bandpass filtered (500–5000 Hz, multi-channel processor, Alpha Omega) to extract multi-unit activity. Data were visualized and evaluated online using Spike2 software (Cambridge Electronic Design, Cambridge, UK) to locate the best possible site of electrode placement directly within the STN (see Figure 2A). The STN was identified by the appearance of elevated background activity together with irregular high-frequency firing interspersed with bursting discharges that were clearly different from the low threshold spike bursts encountered en route in the thalamus. A sharp decline of background neuronal activity together with the absence of spike discharges marked the ventral STN border and the transition to subjacent capsular fibers. Planning coordinates (+5 mm AP, ±2.2 mm ML relative to the interaural line and –8 mm DV relative to dura) were adjusted to match the results of microelectrode-guided delineation of STN boundaries. HFS-electrodes were then inserted with a manual microdrive and fixed onto the skull with dental cement (Heraeus Kulzer; Wehrheim, Germany). Four stainless steel screws (FST, 0.7 mm diameter) were anchored around the trepanations to assure long-term stability of the implant. Two stainless steel recording screws (FST, 1 mm diameter) were placed over the frontal cortex (+11.5 mm AP, ±2 mm ML) for chronic recordings of the electrocorticogram (ECoG). In addition, two reference screws were anchored centrally over the cerebellum (–3 mm AP, 0 mm ML) and over an area of thickened nasal bone (+14 mm AP, 0 mm ML).

### Behavioral Monitoring

To assess the efficacy of 6-OHDA lesions in particular with respect to hypo- and bradykinesia and the influence of prolonged unilateral STN-HFS in parkinsonian and control rats we investigated general locomotor capacities via continuous (23h) monitoring of spontaneous behavior. Time points of behavioral recordings were five days prior and 22±8 days after SNc surgery, as well as directly before and during STN-HFS 18±5 days after electrode implantation (see Figure 1). As described previously [33], we used a video-based tracking system (VideoMot 2.0, TSE Systems, Bad Homburg, Germany) to record movement paths as time series of x-y-positions at a sampling rate of 12.8 Hz. A recording arena (70×100 cm) with three pellet feeders and one water-outlet in the corners was positioned in a custom-made recording box equipped with foamed plastic that provided light and acoustic insulation. Recordings took place under constant darkness. We analyzed locomotor behavior by applying algorithms developed to analyze open-field locomotion in rodents [34]. Speed values (cm/s) were calculated on position time series with a moving window of 0.3 s. Separation of rest vs. motion episodes was based on a noise level of 4 cm/s. Using smoothed histograms of logarithmic maximal speed derivative values (log max-SD) we identified two different natural modes of motion in control and PD rats. We further calculated descriptive statistical parameters, i.e., spatial spread (cm) and average max-SD (cm/s), on episodes of full motion to assess the hypo- and bradykinetic state of PD rats and to compare it with control animals.

### Electrophysiology

To assess oscillatory activitiy along the cortex-STN axis in awake and unrestrained animals during quiet rest approximately 14 days after lead implantation we recorded LFP from STN leads (tip impedance 80-120 kΩ, ring ∼1 kΩ) and ECoG signals from screws implanted above the frontal cortex (see Figure 1). Signals were referenced against nasal and cerebellar screws, pre-amplified, amplified, A/C-coupled and bandpass filtered (1–100 Hz, multi-channel processor, Alpha Omega) and recorded with a sampling rate of 1 kHz (Spike 2). We performed subsequent offline processing steps with routines written in MATLAB (The Mathworks, Natick, USA). These included creation of bipolar derivations of STN-LFP signals and digital notch filtering to remove 50 Hz line noise. Power spectra of LFP and ECoG signals across data epochs of 10–20 min length were calculated using Welch’s method as implemented in MATLAB. For statistical comparisons we normalized the power spectrum density (PSD) to the total power between 1–80 Hz. For illustration purposes we first multiplied power values with the corresponding frequency and then normalized the resulting values to the total power in time-frequency plots to account for the 1/f decay of spectral power. We assessed statistically significant power differences between PD rats and controls within seven frequency bands (delta 1–3 Hz, theta 4–7 Hz, alpha 8–12 Hz, low beta 13–24 Hz, high beta 25–35 Hz, low gamma 36–49 Hz, high gamma 51–80 Hz) using the non-parametric Wilcoxon rank sum test (P<0.007, Bonferroni-corrected for seven frequency bands).

### High-Frequency-Stimulation

At 18±5 days after electrode implantation (see Figure 1), individual animals received continuous unilateral STN-HFS for 23 hours. Awake rats were placed in the monitoring box and stimulation leads were fed out via an electric slip ring allowing free rotation of cables, thus ensuring unrestrained stimulation and monitoring conditions. To generate stimulation, we used a constant current source (Otoconsult, Frankfurt, Germany) with driving input from a waveform generator (Tektronix, Beaverton, USA) and monitored the output stimulus waveform using a digital oscilloscope (Fluke, Glottertal, Germany). HFS-parameters were adapted for safe and chronic application in rodents [31], [32], [35]. Cathodic, bipolar, charge-balanced pulses of 60 μs pulse-width, 130 Hz stimulation frequency and 300 μA current amplitude were applied. We controlled for the current spread to adjacent neuronal structures by carefully monitoring stimulation-induced behavioral responses. As such, dyskinetic movements of the contralateral paw, face or rotational behavior were observed upon a stepwise current increase, consistent with previously reported side effects of stimulation in the subthalamic area [31], [36]. The current amplitude for subthreshold HFS was then set at 300 μA, a value approximately 30% below the threshold for stimulation-induced side effects (450±50 μA, median±MAD).

### Tissue sampling

Immediately after STN-HFS, we induced deep anesthesia with isoflurane followed by i.p.-injection of ketamine (100 mg/kg) and xylazine (6 mg/kg). The time between induction of anesthesia and tissue isolation was crucial with respect to the effect of biological RNA degeneration and the effect of anesthesia itself. We perfused animals transcardially via the ascending aorta with ice-cold saline solution (0.9% NaCl, 10.000 IU heparin added for anti-coagulation; B. Braun) and rapidly removed the brain under ongoing perfusion, thereby cooling down the whole animal body within 5 minutes after induction of anesthesia. The brain was then transferred to a brain slicing matrix (David Kopf Instruments) to cut out a coronal slice ranging from –3 to –6 mm relative to the frontal apex. From this slice, three tissue samples from motor cortical areas M1-2 and S1 of both hemispheres were manually dissected with a sample corer (2 mm diameter; WPI, Sarasota, USA). Probes were immediately flash-frozen in liquid nitrogen and stored at –80°C for mRNA extraction. The second tissue block containing STN, VTA and SNc was immersion-fixed in 4%-PFA-solution (paraformaldehyde in 0.1 M phosphate buffered saline, Sigma-Aldrich) for subsequent histological analysis. In total, the whole procedure lasted less than 15 minutes. Regarding RNA degradation, assessment on the BioAnalyzer-platform (Agilent Technologies, Santa Clara, USA) revealed good quality and high purity of all RNA samples (A_260_/A_280_-ratios 2.06±0.04; data not shown). Brains were removed under ongoing perfusion in an attempt to elaborate the washout process and minimize sample contamination with systemically active anesthetics. Nevertheless, given the fact that other studies showed significant alteration of gene expression after brief exposure of the brain to central anesthetics (ketamine [37] and gamma-hydroxybutyrate [38]), we cannot completely exclude a possible bias.

### Counting of TH-positive cells in the SNc and VTA

To evaluate dopaminergic cell loss in PD rats and controls we performed free-floating tyrosine-hydroxylase (TH) immunohistochemistry on serial 40 μm coronal sections of the midbrain. The immersion-fixed tissue block containing the SNc was transferred to 30%-sucrose solution and kept at 4°C for 24 hours. Sections were cut with a freezing-microtome (Leica Instruments, Wetzlar, Germany) and stained in free-floating fashion for TH-activity. Briefly, sections were washed in phosphate buffer (0.01 M PBS, Sigma-Aldrich), incubated with 3%-H_2_O_2_-solution for three minutes to block endogenous peroxidase activity and incubated with 2% normal horse serum (added with 0.3% Triton X-100, Sigma-Aldrich) for 30 minutes. Sections were then incubated over night at 4°C with the primary TH-antibody (1:250, monoclonal mouse antibody, Novocastra reagents, Leica Microsystems, Wetzlar, Germany), followed by the biotinylated secondary antibody (1:400, Novocastra reagents) for 30 minutes, and afterwards incubated with avidin and biotinylated horseradish peroxidase (ABC kit, Novocastra reagents) for another 30 minutes. TH was visualized by adding peroxidase substrate (0.02% DAB reagent in 0.003% H_2_O_2_ in PBS) for 2 to 10 minutes duration. Finally, sections were mounted on glass slides, dehydrated in an increasing alcohol row and fixed under a cover slid with Roti Histokitt II (Carl Roth, Karlsruhe, Germany).

Unbiased stereological counting of TH-positive cells in the SNc and VTA was performed using Stereoinvestigator 10.0 Software (MicroBrightField Inc., Williston, Vermont, USA) mounted to an Olympus Bx61 brightfield microscope (Olympus Deutschland GmbH, Hamburg, Germany) equipped with a Microfire TM A/R camera (Optronics, California, USA) and an x-y-z galvano table (Carl Zeiss AG, Jena, Germany). The optical fractionator probe [39], [40] was applied on series of 40 μm thick coronal sections. Technical restrictions (preservation of material for molecular processing) did not allow the analysis of the total volume of SN and VTA. In order to sample comparable parts of the SN and VTA, the stereological analysis was centered on an independent anatomical hallmark, chosen as the rootlets of the oculomotor cranial nerve. Overall we sampled six sections (sampling rate of three sections), thereby spanning 600 μm in the cranio-caudal dimension. However, due to availability of continuous TH-sections containing SNc and VTA, two animals were investigated using sampling rates of two and six sections, respectively. Using a 2,5x lens with NA 0.075 we defined three anatomical regions of interest (ROI), i.e., the substantia nigra pars compacta (SNc), substantia nigra pars reticulata (SNr) and ventral tegmental area (VTA) [30]. For anatomical 3-D reconstructions of ROIs and counted cells see Figure S1. Cell counting was performed using a 40x Plan-Neofluar dry type objective lens with NA 0.75 (Carl Zeiss AG) within the SNc and VTA-ROIs. The counting frame (50×50 μm) with a dissector height of 20 μm was applied with a uniform random sampling grid of 150×150 μm (optical dissector volume of 50000 μm^3^, sampling grid area of 22500 μm^2^, [41]). Schmitz-Hof’s second coefficient of error ranged between 0.1 and 0.15 for the VTA of both controls and PD animals, as well as the SNc of controls. For SNc-ROIs of PD animals it ranged between 0.4 and 0.8, thus reflecting the scarceness of TH-positive cells [40], [41]. The total volume of the sampled SNc and VTA parts was stereologically estimated using the Cavalieri method [42]. As our histological regime did not allow sampling the whole SNc and VTA in the cranio-caudal dimension, we expressed TH-positive cell numbers as cell density (cell count/mm^3^) within the sampled volume. Cell densities were calculated for each ROI and hemisphere and then averaged across hemispheres (n = 6 hemispheres per group). We used non-parametric statistical measures for comparing TH-positive cell depletion between PD and control animals (Wilcoxon rank sum test).

### Verification of Electrode Placement and Stimulation Induced Tissue Damage

Placement of electrode leads and the surrounding tissue damage was verified in photomicrographs of serial 40 μm coronal brain sections, cut as described above and stained with cresyl-violet (Nissl 5%-solution, in acetate buffer of pH 3.8−4.0). Sections were mounted on glass slides and incubated with Nissl solution until the desired staining intensity was obtained. Afterwards, sections were clarified in chloroform containing solution, degreased in successively increasing alcohol concentration and fixed as described above for TH-sections. We considered the placement of electrodes as correct only if the electrode tract hit the STN in three consecutive sections. The tissue response to electrode placement and stimulation-induced damage was analyzed in the section showing greatest tissue reaction [43]. The area infiltrated with mononuclear cells (MNC) as defined under light microscopy was determined using commercial image-analyzing software (AxioVision, Carl Zeiss) and compared between pulsed and unpulsed electrodes for statistically significant differences using the non-parametric Wilcoxon rank sum test. MNC are know to infiltrate the sheath of tissue surrounding also unpulsed electrodes [44]. Furthermore, sections were checked for signs of neuronal damage by electrical lesions (i.e., coagulation).

### Gene Expression Profiling

Total RNA was extracted from tissue samples of six STN-HFS treated rats (PD n = 3, control n = 3) using the RNeasy Lipid Tissue Mini Kit (Qiagen, Hilden, Germany), which purifies all RNA molecules longer than 200 nucleotides providing enrichment for mRNA. After quality assessment of RNA (BioAnalyzer-platform, Agilent Technologies, Santa Clara, USA), we analyzed 4 μg RNA extract of two PD and two control rats on Affymetrix Rat Expression 230 2.0 chips (Affymetrix, Santa Clara, USA) as described previously [45]. This required cDNA synthesis, labeling and hybridization according to the protocol of the manufacturer (Affymetrix GeneChip Expression Analysis Technical Manual). Following incubation in the Affymetrix Hybridization Oven 640 at 45°C for 16 h, gene chips were washed and stained using the Affymetrix Fluidics Station 450. We scanned microarrays with the Affymetrix GeneChip Scanner 7G, and processed the signals using GCOS (V1.4, Affymetrix) and Expression Console (V1.1, Affymetrix) software. Chips were scaled to a target value of 300 and normalized using robust multiarray averaging (RMA). We requested uniform quality of MA experiments assessed on the basis of the number of present probe sets, RMA signal histograms, unsupervised hierarchical clustering and Pearson’s correlation matrix of RMA signals. To reduce the influence of systematic noise caused by, e.g., circadian rhythmicity [46] or the behavioral state of the animal [47], our protocol utilized an intra-animal contrast of gene regulation. The first analysis specifically addressed the HFS-effect and incorporated four comparisons of stimulated vs. non-stimulated hemispheres (two intra-animal and two inter-animal cross-comparisons). We generated gene regulation lists with the ‘change p value algorithm’ of the GCOS software package (Affymetrix) which provides change calls on the basis of paired t-tests between corresponding probe sets (n = 31043, Affymetrix Rat Expression 230 2.0). To account for the low number of biological microarray replications we requested from a candidate gene to show equal regulation calls in all four comparisons. In our second analysis, we addressed the gene expression changes that constituted the baseline of our first analysis and may reflect 6-OHDA related changes. To this end, the non-stimulated hemispheres of PD vs. those of control animals were analyzed for homonymous change calls. Regulated transcripts were filtered with a signal-log ratio (slr) threshold of ±0.6 (i.e., the base 2 logarithm of a 1.5 fold-change) for all individual comparisons. Regulation lists were analyzed for functional enrichment of Gene Ontology (GO)-terms and KEGG (Kyoto Encyclopedia of Genes and Genomes) pathways using the DAVID database for annotation, visualization and integrated discovery [48], [49] (http://david.abcc.ncifcrf.gov).

### Real-time RT-PCR analysis

To validate microarray expression profiling for selected genes, real-time RT-PCR analysis was performed as described previously [50]. We report data from validated TaqMan-Gene expression assays for RT1-Da (Rn01427980_m1), Cd74 (Rn00565062_m1), Rasgrp2 (Rn00570056_m1) and Rpl13a (Rn00821946_g1). Four additional genes from a less strict generation of regulation lists (i.e., 2/4 change count, intra-animal comparisons; Gabra3, Glra2, Nxph3 and Syt4) were investigated for exploratory reasons (data not shown). All genes were investigated in triplets. The relative expression of target mRNA was normalized to the housekeeping gene Rpl13A. Change calls were expressed with the ΔΔCT-method (i.e., the base 2 logarithm of the fold change and equals the slr). Within and across group statistical comparisons utilized the non-parametric Wilcoxon rank sum test (fixed effect analysis, P<0.007, Bonferroni-corrected for seven investigated genes).

### Western blot analysis

Proteins were purified from the phenolic phase of the RNeasy Lipid Tissue Mini Kit (Qiagen) as up to 98% of total protein can be solubilized from TRIzol treated samples [51]. To this end the phenolic phase was incubated with 1,5 ml isopropanol (Sigma-Aldrich) for 10 min at room temperature. After centrifugation (11500 x g, 4°C) the pellet was washed three times with 2 ml guanidinhydrochlorid solution (0.3 M in 95% ethanol; Sigma-Aldrich) at room temperature for 20 min each and spun down (4500 x g, 4°C). Finally, the pellet was washed 20 min with 2 ml ethanol (100%, Sigma-Aldrich) at room temperature and airdried after centrifugation. Protein pellets were solubilized in 200 μl reducing protein solving buffer (Machery-Nagel, Düren, Germany), boiled for 10 minutes, centrifuged and supernatants were stored at –20°C for further usage. Western blotting was carried out essentially as described previously [50]. We used polyclonal antibodies against CD74 (C-16; goat-antiserum; dilution 1∶500), RT1-D (OX-17; mouse-antiserum; dilution 1∶200) and RT1-B (HIS-19; mouse-antiserum; dilution 1∶200), from Santa Cruz Biotechnology Inc. (Heidelberg, Germany). Additionally, we used two antibodies to detect the astroglial marker GFAP (GA-5; mouse-antiserum; dilution 1∶1000) and the microglial and macrophage marker Iba-1 (C20; goat-antiserum; dilution 1∶1000). Bound antibody was stained using goat anti-mouse, goat anti-rabbit or rabbit anti goat secondary antibodies conjugated with horseradish peroxidase (Jackson Immuno Research Europe Ltd., Suffolk, UK) and visualized using enhanced chemiluminescence substrate (Pierce Protein Biology Products, Thermo Scientific, Bonn, Germany). X-ray films of Western blots were scanned and protein bands were quantified by densitometry using an ImageJ plugin (Version 1.46, NIH, USA).

### Statistical analysis

Throughout the paper, we utilized non-parametric statistical measures to check for significant differences between PD and control animals (Wilcoxon rank sum test). However, generation of regulation lists using GCOS (Affymetrix) applied paired t-tests between corresponding probe sets, as implemented in the software package. Statistical results are presented as mean±standard deviation (SD) unless stated otherwise. The alpha level of <0.05 was corrected for multiple comparisons using Bonferroni’s method in case of power-spectral and RT-PCR analyses.

## Results

### Verification of HFS electrode placement and tissue reaction

Microelectrode-guided targeting of the STN led to a high rate of successfully implanted STN-HFS electrodes in rats used for gene expression profiling (n = 6; for a typical electrode trajectory, see Figure 2A). In 10/12 implanted hemispheres we detected a hit of the STN in at least three consecutive sections. One control rat was implanted approximately 250 μm anterior to the rostral margin of the STN. However, as the threshold for inducing typical dyskinetic movements in this animal did not differ from the group average, it was still included in confirmatory RT-PCR validation. Stimulated and non-stimulated hemispheres exhibited mild MNC infiltration, which was expected as a reaction to the electrode implantation itself [44] (see Figure 2B). Non-parametric statistical comparison revealed a trend but no significant difference between pulsed and unpulsed electrodes (MNC infiltrated area around pulsed electrode tips: 1.79±0.3×10^5^ μm^2^ vs. 1.14±0.7×10^5^ μm^2^ around unpulsed tips; mean±SD, P = 0.065, Figure 2C). Additionally, two stimulated hemispheres (1 PD, 1 control) showed small areas of coagulation (1.3 and 1.7×10^5^ μm^2^). Cortical tissue from those animals was not used for microarray screening, but included in confirmatory RT-PCR analysis.

### Stereological quantification of TH-positive cells

**Figure 3:**
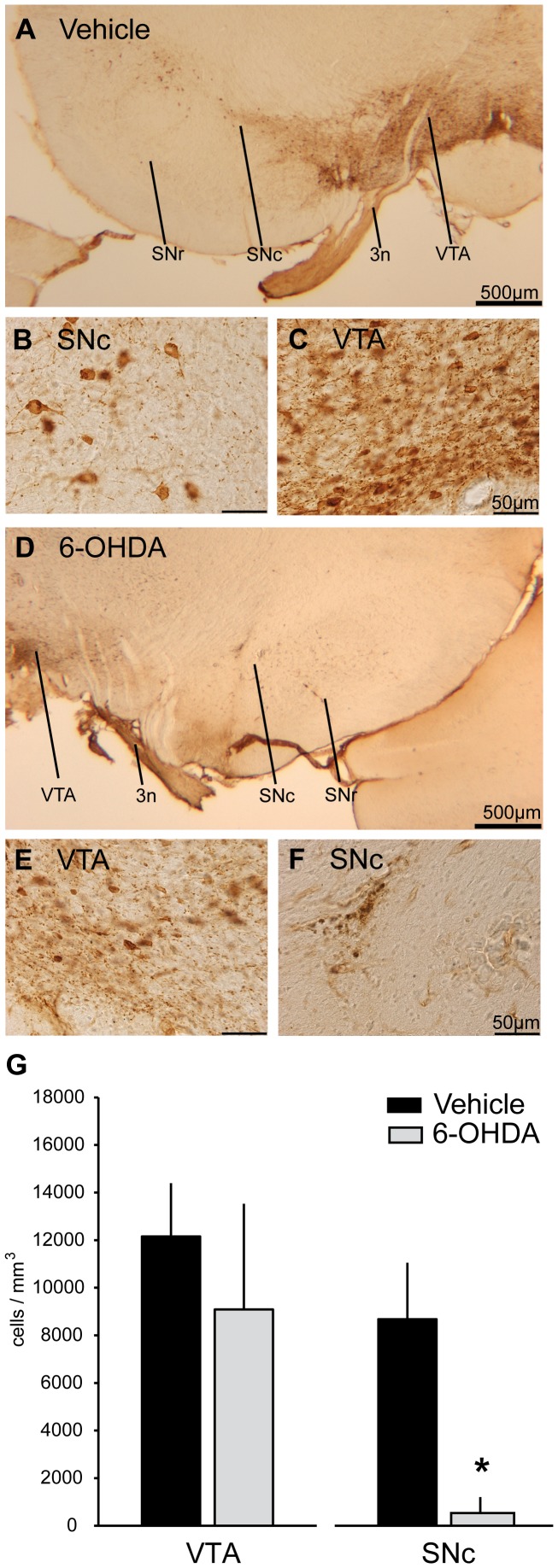
Stereology of tyrosine-hydroxylase positive midbrain neurons. Representative sections (2,5x lens) of the midbrain depicting the rootlets of the third oculomotor cranial nerve (3n), VTA, SNc and SNr stained for tyrosine-hydroxylase (TH) of an example control (A) and example PD rat (D). Photomicrographs show magnifications taken with a 40x lens and depict TH-positive dopaminergic cells in the SNc (B) and VTA (C) of a vehicle injected rat, as well as of a 6-OHDA injected rat (E and F, respectively). Results of unbiased stereological cell counting (G) are presented as estimated cell density (mean±SD cells/mm^3^) for the VTA and SNc of all rats included in gene expression profiling (n = 3 PD, n = 3 control). Filled black bars indicate cell estimates respectively of vehicle and gray filled bars of 6-OHDA injected rats. Statistically significant differences are marked with an asterisk. 3n, 3rd oculomotor cranial nerve; VTA, ventral tegmental area; SNc, substantia nigra pars compacta; SNr, substantia nigra pars reticulata; TH, tyrosine-hydroxylase; 6-OHDA, 6-hydroxydopamine.

Bilateral injections of 15 μg 6-OHDA into the SNc resulted in extensive cell death of dopaminergic neurons in the SNc approximately 14 weeks after neurotoxin injections (see Figure 3A-F for representative photomicrographs of the midbrain at approximately –5.5 mm relative to Bregma of one control and one PD rat; see Figure S1 for 3-D reconstructions of stereological results). The cell density of nigral TH-positive neurons was reduced by –94% in PD animals compared to controls (532.3±672.9 cells/mm^3^ in PD vs. 8675.6±2374.5 cells/mm^3^ in controls; P = 0.002; Wilcoxon rank sum test; see Figure 3G). VTA neurons exhibited no significant depletion (9084.4±4441.3 cells/mm^3^ in PD vs. 12156±2233 cells/mm^3^ in controls, –25.3%, P = 0.3; see Figure 3G). Comparison of depletion values between single hemispheres yielded similar values for both hemispheres except for one PD animal that showed depletion of VTA dopaminergic neurons in the right hemisphere (data not shown). The cell density of SNc neurons of vehicle injected rats was comparable to numbers of TH-positive cells reported by a previous study [52] that carried out investigations in Fisher 344XBrown Norway hybrids. Other studies reported significantly higher values in different rat strains (Lewis rats [53], Long-Evans rats [54], Sprague-Dawley rats [55]), which suggests large differences in the absolute number of mesolimbic dopaminergic cells in different rat strains.

### Behavioral monitoring revealed hypo- and bradykinetic state

Upon visual inspection, spontaneous locomotion and movement speed of 6-OHDA-injected rats appeared reduced and body posture revealed a hunchback-like shape. Hind limb rigidity was detectable upon manual assessment. PD rats displayed anorexia and adipsia to a variable degree, leading to an average postoperative weight loss of 17.2±9.2% vs. 1.1±3.7% (mean±SD) in controls prior to behavioral monitoring (P = 0.13). No control rat showed overt bradykinesia, anorexia or adipsia. Nine of twelve PD rats stabilized weight loss within 14 days after SNc surgery. No additional signs of altered animal behavior that could indicate persistent pain distress, e.g., vocalization upon gentle palpation or mutilation such as licking, biting, scratching or other form of harming body parts was detected during daily inspections. In synopsis of all clinical features we interpreted the presented behavior as a pronounced parkinsonian phenotype following bilateral 6-OHDA injections into the SNc.

**Figure 4:**
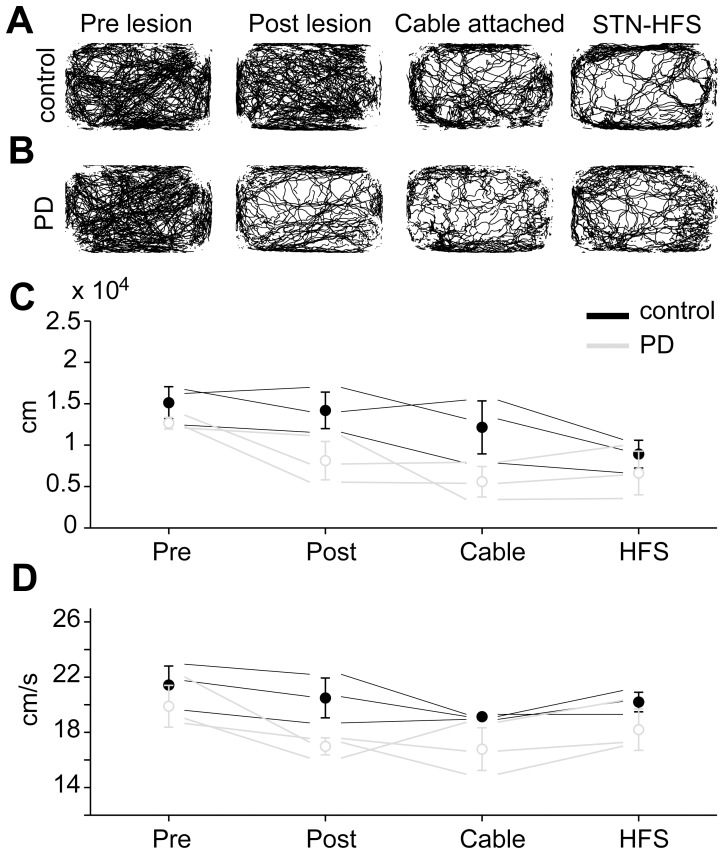
Spontaneous locomotor activity revealed parkinsonian phenotype. The upper row (A) depicts spontaneous 23h locomotor activity of a representative control rat, the lower row (B) of an example PD rat. Recordings were performed before (left) and after 6-OHDA or vehicle injections (left-middle) as well as before (right-middle) and during unilateral STN-HFS (right). The lower row depicts descriptive statistics of spatial spread (C) and maximal movement speed derivative (max-SD; D) averaged over episodes of full blown motion for all four recording times (see above). Markers and error-bars indicate the mean±SD of animals included in gene expression profiling (n = 3 each group; controls filled-black, PD rats open-gray symbols). Thin lines indicate each single animal included in gene expression profiling. PD, Parkinsońs disease; 6-OHDA, 6-hydroxydopamine; STN, subthalamic nucleus; HFS, high-frequency stimulation.

Quantitative assessment of the absolute spatial spread and the maximal speed derivative (max-SD) obtained from episodes of full blown motion confirmed the reduced locomotor activity of PD vs. control rats included in gene expression experiments. Figure 4A-B shows examples of raw movement tracks of one control (A) and one PD animal (B) at the different time points of behavioral monitoring. Postoperatively, the spatial spread was decreased on average to 81.3±28.4 m in PD rats vs. 142±27 m in controls (see Figure 4C). The max-SD was reduced to 17±0.7 cm/s in PD rats vs. 20.5±1.8 cm/s in controls (see Figure 4D). Compared to preoperative values, PD rats showed a relative reduction of –35.7% for spatial spread and –14.6% for max-SD in contrast to –6.2% for spatial spread and –4.3% for max-SD of controls. Note that spatial spread and max-SD values of rats included in gene expression experiments were placed well within the group variance at pre- and postoperative recording times (n = 9 PD and n = 8 controls, data not shown). However, the obtained pre- and postoperative behavioral estimates did not differ significantly in a non-parametric statistical comparison (P = 0.1 for PD pre vs. postoperative; P = 1 for controls pre vs. postoperative; n = 3 PD and n = 3 controls).

To assess the reversal of experimental parkinsonism by 23h of unilateral STN-HFS, we monitored PD and control rats before (cables attached, no stimulation) and during 130 Hz stimulation. Notably, STN-HFS in PD rats led to an increase of 18.9% in spatial spread compared to cable attached monitoring in the absence of stimulation. In contrast, control animals showed a decrease of –26.7% in response to STN-HFS (see trend in Figure 4C-D). Although observable in raw movement tracks (Figure 4A-B, middle-right and right inserts) these trends likewise did not reach statistical significance. The max-SD showed a mild and non-significant increase of 8.5% in PD and 5.5% in control rats due to STN-HFS.

### Pronounced oscillatory activity in STN- and ECoG-LFPs

**Figure 5:**
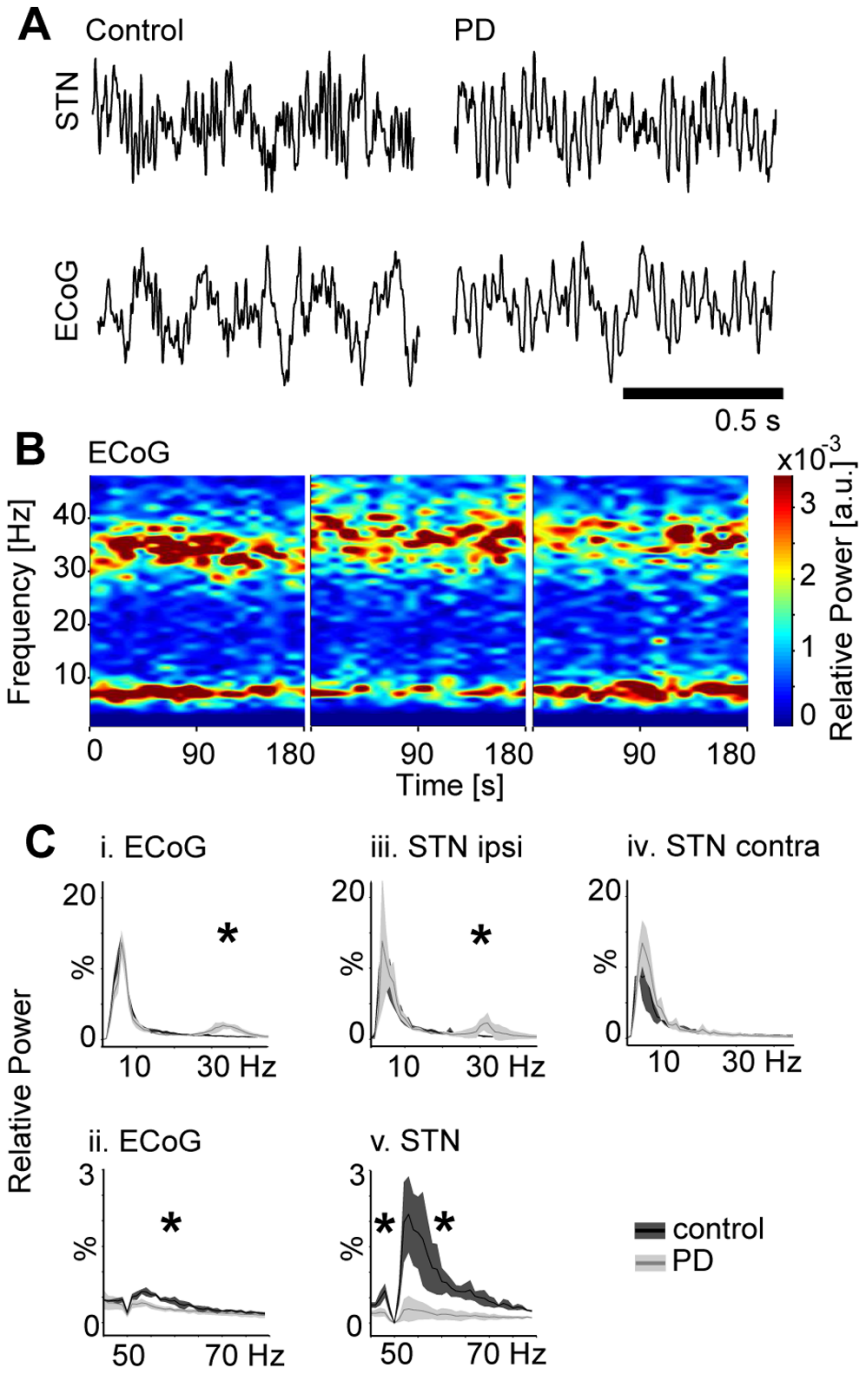
Spectral analysis of cortical and subthalamic local field potentials. Representative examples (A) of oscillatory activity in the raw local field potentials (LFP) recorded from STN (upper row) and frontal ECoG (lower row) of controls (left column) and 6-OHDA treated parkinsonian rats (right column). Time-frequency plots (B) of spectral power during 180 s of quiet rest from all PD animals included in gene expression analyses. Power spectra (C) calculated from frontal ECoG and STN-LFP data of parkinsonian rats (light gray lines/shadings) and controls (dark gray lines/shadings): (i) low-frequency range (1–45 Hz) and (ii) high-frequency range (45–80 Hz) of ECoG-power spectra pooled across all hemispheres (n = 6). (iii) Low-frequency power spectrum of STN-LFP ipsilateral to the side of HFS and (iv) contralateral to the side of HFS (n = 3 each). (v) High-frequency power spectrum of STN-LFP pooled across all hemispheres. Asterisks indicate significantly different frequency bands. Note the dampening at 50 Hz that resulted from bandpass filtering of line noise. LFP, local field potential; STN, subthalamic nucleus; ECoG, electrocorticogram; 6-OHDA, 6-hydroxydopamine, HFS, high-frequency stimulation.

Oscillatory activity in the 30–40 Hz-frequency range was clearly observable in monopolar frontal cortex ECoG and bipolar STN-LFP recordings in PD rats (6/6 and 3/6 hemispheres, respectively), but not in controls (0/6 and 0/6, respectively). Figure 5A depicts short epochs of raw STN-LFP (upper row) and frontal cortex ECoG (lower row) data recorded in PD rats (right column) and controls (left column) during quiet rest. Figure 5B shows time-frequency plots of LFP- and ECoG-power of all PD animals included in gene expression profiling. We saw marked oscillatory activity in the STN of 3/6 PD-hemispheres and chose the side of HFS accordingly, taking into account that oscillatory strength and extent of the oscillatory region in PD patients correlates with DBS-efficacy [56]. We calculated grand average power spectra (Figure 5C) by pooling data across all hemispheres of all animals included in gene expression profiling and, in case of STN-LFPs, by pooling separately across hemispheres ipsi- and contralateral to HFS electrodes. We compared the relative power in seven different frequency bands between PD rats and controls using non-parametric statistics (significant frequency bands are labeled with an asterisk in Figure 5Ci-v; P<0.007, Bonferroni-corrected for seven frequency bands). In ECoG recordings, relative power in the high beta/low gamma band (25–34 Hz) was significantly increased in PD rats compared to controls (Figure 5Ci). A less broad but similarly distinct and significant peak was visible in the grand average of STN-LFPs (Figure 5Ciii) ipsilateral to subsequent HFS. No peak was visible on the contralateral side (Figure 5Civ). At the same time, relative power in the gamma range (35–47 and 51–80 Hz) was significantly decreased in PD vs. controls. This decrease in gamma-power was most pronounced in STN-LFPs (Figure 5Cv), but also present in ECoGs (Figure 5Cii) recorded from PD rats.

### STN-HFS induced changes in sensorimotor cortical gene expression

STN-HFS led to downregulation of six genes in PD rats showing homonymous regulation in both intra- and across-animal comparisons (see Table 1). Five genes encoded major histocompatibility complex (MHC) class II proteins (i.e., RT1 class II, locus Ba with three transcripts, locus Bb, locus Da and locus Db1, Cd74 antigen) and one gene encoded an MHC-class I protein (RT1 class I, CE5). These genes comprised a significantly enriched cluster of ‘Antigen processing and presentation of peptide antigen’ related genes (EASE = 5.3; the EASE-score is the minus log transformation of annotation P-values, [40]). Furthermore, they also constituted the members of ten significantly enriched KEGG-pathway, e.g., ‘Antigen processing and presentation’ and ‘Cell adhesion molecules (CAMs)’. Along with MHC genes the Complement factor 2 gene (C2) was downregulated in three of four comparisons, enlarging the group of immune response genes in our data set. Notably, none of these immune response genes was regulated by STN-HFS in vehicle injected controls.

**Table 1:** STN-HFS regulated MHC genes in sensorimotor cortex.

			STN-HFS effect in 6-OHDA group			STN-HFS effect in vehicle group			Lesion effect		
Gene Title	Gene Symbol	Probe Set ID	D-count	I-count	slr	D-count	I-count	slr	D-count	I-count	slr
RT1 class II, locus Da	RT1-Da	1370883_at	4	0	–2.7	0	0	—	0	4	1.5
RT1 class II, locus Db1	RT1-Db1	1370383_s_at	4	0	–2.7	1	1	—	0	4	1.9
RT1 class II, locus Bb	RT1-Bb	1371033_at	4	0	–2.6	1	0	—	0	4	1.5
CD74 antigen	Cd74	1367679_at	4	0	–2.4	0	0	—	0	3	1.2
RT1 class II, locus Ba	RT1-Ba	1381593_x_at	4	0	–2.4	0	0	—	0	4	1.2
RT1 class II, locus Ba	RT1-Ba	1392334_at	4	0	–2.2	0	0	—	0	4	1.3
RT1 class II, locus Ba	RT1-Ba	1370822_at	4	0	–1.6	0	0	—	0	4	1
RT1 class I, CE5	RT1-CE5	1388255_x_at	4	0	–1.4	1	0	—	0	0	—

List of all microarray regulated genes exceeding the slr-threshold of ±0.6 and showing homonymous regulation in both intra- and across-subject comparisons. Gene name, gene symbol and Affymetrix ID are given with the respective decrease (D) or increase (I) count as well as the mean slr. Data are presented for 6-OHDA and vehicle treated rats and for the effect of lesion analysis that compared the gene expression change between the non-stimulated hemispheres of 6-OHDA and control rats.

Slr, signal-log ratio; 6-OHDA, 6-hydroxydopamine.

All MHC class II genes, but not the MHC class I gene or C2, were found upregulated in 6-OHDA lesioned rats (lesion effect analysis). That is, the slr-change direction due to STN-HFS was opposite to the slr-change direction of the lesion effect comparison. Hence, increased expression of MHC class II genes observed in parkinsonian rats following 6-OHDA lesioning was reversed by STN-HFS (see Table S1). We termed this effect ‘counter-regulation’.

### Microarray confirmation with RT-PCR and Western blotting

**Figure 6:**
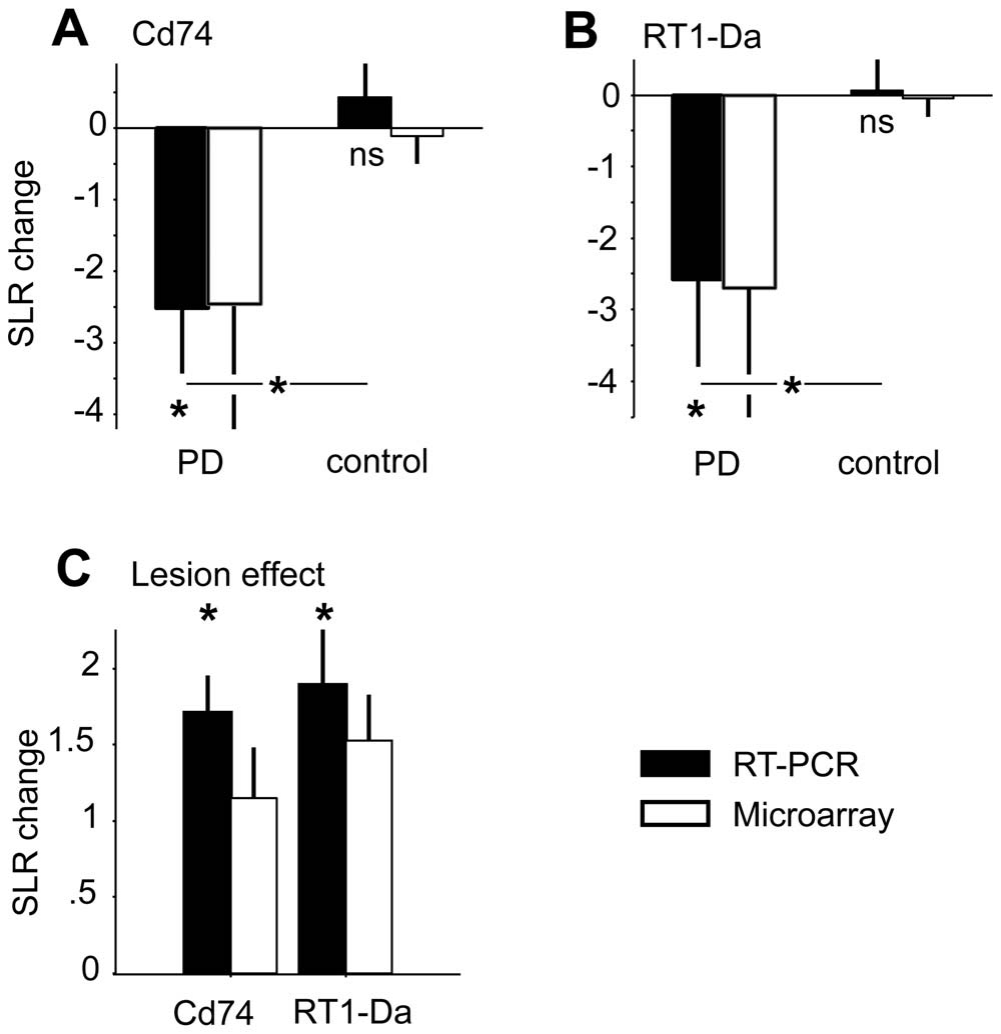
Real-time RT-PCR. Real-time RT-PCR results for MHC class II genes Cd74 (A) and RT1-Da (B) regulated by unilateral STN-HFS. Lesion effect comparison (C) for both MHC class II genes. Filled black bars indicate signal-log changes of RT-PCR analysis and open bars the results of microarray experiments (mean±SD). Asterisks denote statistically significant results. MHC, major histocompatibility complex; STN, subthalamic nucleus; HFS, high-frequency stimulation.

RT-PCR analysis and Western blotting were based on samples from a total of three 6-OHDA and three vehicle injected animals (i.e., aliquots of the same two samples per group that had been used for microarray experiments plus RNA isolated from an additional animal per group). MHC class II genes Cd74 and RT1-Da showed significant homonymous downregulation in RT-PCR analysis, not only confirming microarray results but also revealing a similar strength of expression changes in RT-PCR (see Figure 6A-B). Comparable to the microarray lesion effect analysis, Cd74 and RT1-Da showed upregulation by 6-OHDA injections in confirmative RT-PCR comparisons of the non-stimulated hemispheres (Figure 6C).

Western blotting revealed that the MHC class II protein RT1-D showed downregulation by STN-HFS in all investigated PD rats (see Figure S2). CD74 showed downregulation on the protein level in both PD rats that were investigated with microarrays but not in the third PD rat that had received STN-HFS and was added for confirmatory analyses. For RT1-B, another MHC class II member, we did not observe STN-HFS induced downregulation on the protein level as suggested by microarray analysis. Vehicle injected control animals showed no regulation on the genetic level and no consistent regulation pattern on the protein level due to STN-HFS. To investigate whether the changes in immunity-related genes and proteins were associated with an altered state of activity in astrocytes or abundance of microglial cells or macrophages we additionally investigated the protein levels of GFAP and Iba-1, respectively. GFAP protein regulation resembled the protein regulation pattern of CD74 in both 6-OHDA-lesioned and control animals (see Figure S2 and S3). In contrast, only low levels of Iba-1 protein could be detected without revealing a distinct regulation pattern.

## Discussion

Dopamine depletion in PD leads to a wide range of pathological alterations of the cortico-basal ganglia network, ranging from gene expression to network synchrony that ultimately lead to motor symptoms. The therapeutic effects of STN-HFS on different motor symptoms occur at variable latencies, which likely reflects the multiple effects on these different levels of organization. Here we examined HFS-induced chances in gene expression in control and 6-OHDA lesioned animals displaying the behavioral phenotype and electrophysiological signatures of PD. The results suggest that the expression of MHC class II genes in sensorimotor cortex is increased by dopamine depletion and returned to baseline levels by STN-HFS. Understanding the complex interplay of MHC genes in PD might eventually help to understand the neural mechanisms underlying the therapeutic mechanism of STN-HFS in this debilitating disease.

### Technical considerations

We chose to apply a whole genome analysis in order to identify genes regulated by STN-HFS in tissue samples taken from rat sensorimotor cortex, as this cortical area is a major source of “hyperdirect” cortico-STN afferents, the importance of which has repeatedly been highlighted in attempts to explain the therapeutic effects of STN-HFS [16], [17], [22]. We deliberately chose to investigate HFS-effects in brain tissue that did not receive direct electrical stimulation, avoiding contamination due to tissue reaction or damage following direct electrical manipulation.

We utilized an intra-animal contrast, comparing stimulated and non-stimulated hemispheres within the same animal. Bearing in mind the almost infinite endogenous and exogenous factors having an effect on gene expression this represents a viable biological control. It is impossible to create exactly the same test conditions for different animals with respect to, e.g., circadian rhythm, diet, light, noise or handling. In fact, a large set of genes (approximately 1–10% of all present transcripts in brain or liver tissue) is known to oscillate in a circadian manner [46] or is influenced by the behavioral state and vigilance of the animal [47]. These circumstances would lead to unspecific noise in an across-animal experimental design with the potential to obscure small but biologically relevant changes in STN-HFS related gene expression. The intra-animal contrast is robust against such factors, as global influences on gene expression should influence both hemispheres similarly and would filter out as a result of the differential analysis. On a behavioral level, unilateral STN-HFS did not alter the motor performance of PD animals with statistical significance**.** Nonetheless, a trend for normalized spatial spread and movement speed was observed. Interestingly there is also clinical evidence for a bilateral motor benefit in PD patients treated by unilateral STN-HFS [57]–[59]. However, compared to the pronounced and persistent contralateral effects typically observed with STN-HFS, ipsilateral benefits are much smaller and show a marked attenuation over time. A recent study [17] investigating STN-HFS evoked cortical potentials only found a small effect in the contralateral hemisphere following stimulation with 60Hz, but not 130Hz, the frequency used in the present study. Consistent with this finding, we did not observe induction of immediate early genes in the contralateral hemisphere after unilateral 130 Hz STN-HFS in a previous study [23]. Taken together, the complex reorganization of cortico-basal ganglia network structures and -functioning will likely involve both ipsi- and contralateral effects of unilateral stimulation. With our experimental design, we can detect genes that exhibit a differential regulation with a high sensitivity but would under appreciate or miss out bilaterally regulated genes. Furthermore, we could underestimate the regulation strength of unequally regulated genes. Thus, to obtain a complete and unbiased perspective on gene regulation under STN-HFS, follow-up experiments that utilize the across-animal comparison with an implanted but not stimulated group of controls would be necessary.

General anesthesia itself alters gene expression in the brain [37], [60], and is a strong modulator of neuronal activity in subcortical structures [61] or of cortical connectivity and network states [38]. Such vigilance-dependent modulation might interfere with the genomic baseline, possibly masking relevant changes induced by STN-HFS. Whereas STN-HFS was applied to the freely moving animal, tissue harvesting took place after brief induction of anesthesia. Although dose-dependent effects of centrally active anesthetics may have been ameliorated by the immediate perfusion with ice-cold saline, we cannot exclude a possible bias. However, as the contralateral side to stimulation served as an internal control, bilateral effects of anesthesia could filter out in our experimental design.

Studies addressing HFS-regulated gene expression often utilize acute stimulation regimes of up to 3 hours. A chronic animal model enables longer stimulation times and allows for a behavioral assessment of stimulation effects and efficacy. We chose an intermediate stimulation time of 23 hours in order to identify genes that are up- or downregulated after an initial phase of immediate early gene induction and are possibly involved in adaptive processes.

Histological analyses revealed mild infiltration of mononuclear cells around both pulsed und unpulsed electrode tips as well as two small electrical lesions in one PD and control rat each. Though distant to the sensorimotor cortex, STN lesions may still bias cortical gene expression. To minimize the rate of false positive genes we excluded the respective samples in exploratory microarray analyses. Thus, minor electrical lesions would not bias the hypothesis that a found candidate gene is regulated by STN-HFS in a biologically meaningful way. In this respect, the homonymous regulation of several genes from a certain gene network represents an immanent biological control.

In order to have matched control hemispheres we used bilaterally lesioned animals, which resemble more closely the extent of dopamine loss in most patients compared to hemi-lesioned animals. In our disease model, dopaminergic depletion of the SNc was nearly total (–94% compared to controls), whereas the VTA exhibited no significant cell loss. This dissociation of dopaminergic degeneration is comparable to that seen in humans with PD, where the VTA is significantly less affected than the SN [62]. However, VTA cell loss was more variable and the alteration of the mesolimbic dopamine system in our model could potentially complicate a differentiation between pure parkinsonian motor symptoms (unambiguous loss of movement and bradykinesia) and the extent of motivational deficits leading to apathy, abulia, or anorexia. Nonetheless, human post-mortem studies have estimated 40-50% cell-loss in the VTA [62]–[66] emphasizing the vulnerability of the mesocorticolimbic dopamine projection in PD. Thus, mesolimbic denervation contributes to the complex clinical phenotype encountered in humans with PD [66], especially in later disease stages.

### Spectral fingerprints of PD

Pronounced oscillations with a peak frequency of 32 Hz were observed along the cortico-STN axis of PD rats, along with a severe reduction of power in the gamma-frequency band (45–80 Hz) compared to controls. To the best of our knowledge, this report is the first to demonstrate excessive high beta/low gamma oscillatory activity in the bilateral 6-OHDA rat model. Enhanced beta-oscillations are a widely accepted biomarker reflecting pathological network activity in akinetic-rigid PD patients [67] and animal models of parkinsonism [68]. The observed high beta/low gamma oscillations in our bilateral lesion model resemble oscillations in hemilesioned rats [69] and exhibit higher frequencies compared to anesthetized rats (peak frequency of 20 Hz [68]). The observed decreases in gamma-power match results obtained from surgically treated PD patients, in which increased gamma-power in the STN-LFP was observed following dopaminergic medication [70]. Thus, bilateral dopamine depletion led to dramatically altered cortical-subcortical dynamics in our animals. This network dysfunction was associated with the appearance of a parkinsonian phenotype on the behavioral level.

### Protein expression of selected candidate genes

We did not find strong similarities between protein abundance and mRNA expression of selected genes (see Figure S3). RT1-D protein levels correlated best with mRNA regulation detected by microarray and RT-PCR analysis**.** Results for the other genes were more inconsistent and in case of CD74 protein levels were detected for single rats that would not have been predicted from transcript levels. At first glance the weak correlation between mRNA and protein levels may appear surprising. However, this finding is supported by ample evidence that the correlation between mRNA and protein levels is highly variable and weak. Recent studies showed that only 30–40% of the protein level can be predicted from mRNA transcription [71]–[75]. Numerous mechanisms regulate the process from transcribing the genetic information until degradation of the protein. This includes mRNA transcription itself, RNA processing (e.g., splicing), RNA stability (half-life differences between transcripts of several hours), influence of regulatory microRNA, regulation of translation (e.g., 3'- and 5'-UTR and other regulatory elements), protein stability (half-life differences from minutes to days) and protein degradation. Therefore there is a weak link between mRNA and protein levels even without taking into account that protein levels alone are bad predictors of protein function in a cellular context. Protein may exist in abundance but may not be fully functional or inactivated (e.g., by phosphorylation, glycosylation and other posttranslational modifications), not translocated to its proper site of action or interacting partners may be missing. Furthermore perturbed systems are more difficult to investigate than steady-state systems, especially when analyzed at a single point in time. For instance, recent upregulation of a given transcript may result in increased protein levels that cannot be detected yet, and vice versa. Similarly, increased transcription may reflect gene induction that was brought about by feedback mechanisms triggered by reduced protein levels. At any rate, RNA synthesis is tightly regulated in cells and depends on a variety of factors. One should keep in mind that mRNA transcription represents the most upstream step and basically the main switch for operating the more or less fixed genetic code. Although proteins bring about phenotypic changes and convey cellular functions, the detection of induced or repressed genes stands for a shift in the currently activated genetic program at the most fundamental level.

### STN-HFS decreases cortical MHC gene expression

The cortical expression of MHC class I and II associated genes was strongly decreased by STN-HFS as detected by microarray and RT-PCR analysis. One of these genes, RT1-D, was strongly downregulated on the RNA level and also showed repressed levels of its protein by Western blotting. Notably, rats rendered parkinsonian by the injection of 6-OHDA revealed upregulation of MHC class II, but not class I genes compared to control animals. Thus, the net effect of STN-HFS on MHC class II gene repression in PD rats was ‘normalization’ to expression levels similar to those of non-parkinsonian controls.

MHC class II genes are expressed by reactive microglia and astrocytes [76], and have been linked to the pathology in human PD [77] and PD animal models [78]. Excessive microglial activation is observed in neural structures that undergo neurodegeneration in PD, in particular substantia nigra, striatum, and globus pallidus [77], but also in hippocampus, limbic structures or cortex of parkinsonian brains [79]. In agreement with our findings, MHC class II genes and Complement factor 3 were found upregulated in the basal ganglia of hemi-parkinsonian rats in a microarray screening study [26]. However, three hours of unilateral STN-HFS did not result in downregulation of MHC class II genes in this study, which investigated a block of tissue that included the site of stimulation (STN) and parts of the basal ganglia, but not cortex. Interestingly, HFS of the ventrolateral thalamus was recently reported to upregulate immune-response related genes in the hippocampus of naïve rats [25]. Another report found a similar set of MHC class II genes (RT1-Ba, -Da and Cd74), MHC class I genes and complement factors (e.g., C2, C3) regulated by HFS applied to the dentate gyrus [80]. In this report HFS in awake but not in anesthetized control rats increased the expression of immune response genes. This suggests that MHC gene regulation, in one way or the other, is altered by electrical high-frequency stimulation of the brain. The change direction, however, would depend on or represent part of the biological consequences of STN-HFS in specific brain structures.

Regarding this, several lines of evidence emphasized the important role that MHC molecules play in neuron-neuron or neuron-glia interactions. MHC class I molecules are involved in synaptic remodeling and establishment of long term potentiation/-depression in the developing neural system [81], [82]. Nonetheless there is evidence that MHC class II expression on glial cells may be tightly coupled to neuronal activity [83], [84]. In contrast to MHC class I regulation, chronic blockade of neuronal action potentials or of neurotransmitter release increased the expression of MHC class II molecules in microglia. Glutamate administration or stimulation of the p75- receptor with neurotrophins reversed the typical absence of MHC class II expression. Hence, MHC class II expression in microglia is regulated by activity-dependent neuronal mediators and reflects the global activity state of a neural structure [85] in a reciprocal fashion: MHC class II expression appears to rise if physiological neuronal activity is diminished. In this context, upregulation of MHC molecules in 6-OHDA lesioned animals may reflect impaired cortical neurotransmission and decreased levels of cortical activity in parkinsonian animals. In turn, STN-HFS induced MHC class II downregulation would be associated with normalization of cortical activity. The finding of a similar regulation of one MHC class II protein (CD74) and GFAP, an astroglial marker that is upregulated in various pathological states and indicates activated astrocytes, is consistent with this hypothesis. On the other hand, no distinct pattern ob Iba-1 regulation could be detected. Hence no excess presence of microglia or macrophages in 6-OHDA treated rats or response to STN-HFS could be shown. In situ studies would be required to determine the effects of STN-HFS on individual cell types in the sensorimotor cortex with respect to transcription and protein expression of MHC-related genes.

## Conclusion

Our central finding is that STN-HFS reversed upregulation of MCH class II genes elicited by 6-OHDA lesioning. Thereby it distinctly influences remote sensorimotor cortical areas at a molecular level, giving rise to adaptive changes that may outlast the immediate and short-term effects of STN-HFS. Future research on immunity-related processes and their interplay with STN-HFS may represent a promising perspective on how pathological cortical activity of the Parkinsonian state may be altered and possibly normalized by STN-HFS..

## Supporting Information

Figure S1 **3-D reconstructions of stereology.** Panels (A-F) depict 3D-reconstructions of stereological regions of interest for all 6-OHDA (left column) or vehicle injected rats (right column) used in gene expression profiling. Different ROIs are color-coded (white: left SNr, pale green: left SNc, purple: left VTA, green: right VTA, pale blue: right SNc, cyan: right SNr). Stereological cell counting was only performed in VTA and SNc-ROIs. Spheres indicate stereologically counted cells in the SNc and VTA. 6-OHDA, 6-hydroxydopamine; ROI, region of interest; SNr, substantia nigra pars reticulata; SNc, substantia nigra pars compacta; VTA, ventral tegmental area.
(TIF)

Figure S2 **Western blot analysis.** Detection of MHC class II candidate proteins (RT1-D, RT1-B, CD74), astroglial (GFAP) and microglial (Iba-1) markers by Western blot analysis. All candidates belonged to the enriched gene cluster ‘antigen processing and presentation of peptide antigen.’ The housekeeping protein tubulin served as biological control. All twelve lanes for a given antigen were blotted simultaneously and cropped later for graphical display. 6-OHDA, 6-hydroxydopamine; HFS, high-frequency stimulation; MHC, major histocompatibility complex.
(TIF)

Figure S3 **Comparison of microarray, RT-PCR and Western blot regulation changes.** Quantification of signal-log ratio (slr) changes of MHC class II candidates (RT1-D, RT1-B, CD74), astroglial (GFAP) and microglial (Iba-1) markers. We utilized densitometric quantification to evaluate protein levels in Western blots. No statistical comparison was applied due to low sample size and missing data regarding RT-PCR (RT1-B, GFAP and Iba-1 were only investigated by Western blotting).
(TIF)

Table S1 **Microarray expression values MHC class II genes.** RMA-normalized microarray expression values of ‘counter-regulated’ MHC class II genes are given for single microarray experiments, respectively for both 6-OHDA and vehicle injected rats. The mean slr of the HFS-effect equals the binary logarithm of the fold change between the not stimulated and stimulated hemispheres of either 6-OHDA or vehicle injected rats. The mean slr of the lesion effect is calculated across groups between the non-stimulated hemispheres of vehicle and 6-OHDA injected rats. Counter-regulation is characterized by opposite regulation direction in the effect of lesion vs. the HFS-effect comparison. That is, STN-HFS normalizes expression values on the stimulated side of PD rats to values of controls on both stimulated and not stimulated hemispheres. RMA, robust multiarray averaging; 6-OHDA, 6-hydroxydopamine; slr, signal-log ratio; HFS, high-frequency stimulation; STN, subthalamic nucleus.
(DOCX)
