# Correction: High-Frequency Stimulation of the Subthalamic Nucleus Counteracts Cortical Expression of Major Histocompatibility Complex Genes in a Rat Model of Parkinson’s Disease

**DOI:** 10.1371/journal.pone.0116365

**Published:** 2014-12-23

**Authors:** The PLOS ONE Staff

An affiliation for the 1st author is missing. Benjamin Grieb is also affiliated with: Clinic of Neurosurgery, University Medical Center Hamburg-Eppendorf, Hamburg, Germany.
