# Supplementary figures and images for: High-Frequency Stimulation of the Subthalamic Nucleus Counteracts Cortical Expression of Major Histocompatibility Complex Genes in a Rat Model of Parkinson’s Disease
Source: PLoS One. 2014 Mar 12;9(3):e91663. doi: 10.1371/journal.pone.0091663 (PMC3951430; doi:10.1371/journal.pone.0091663)

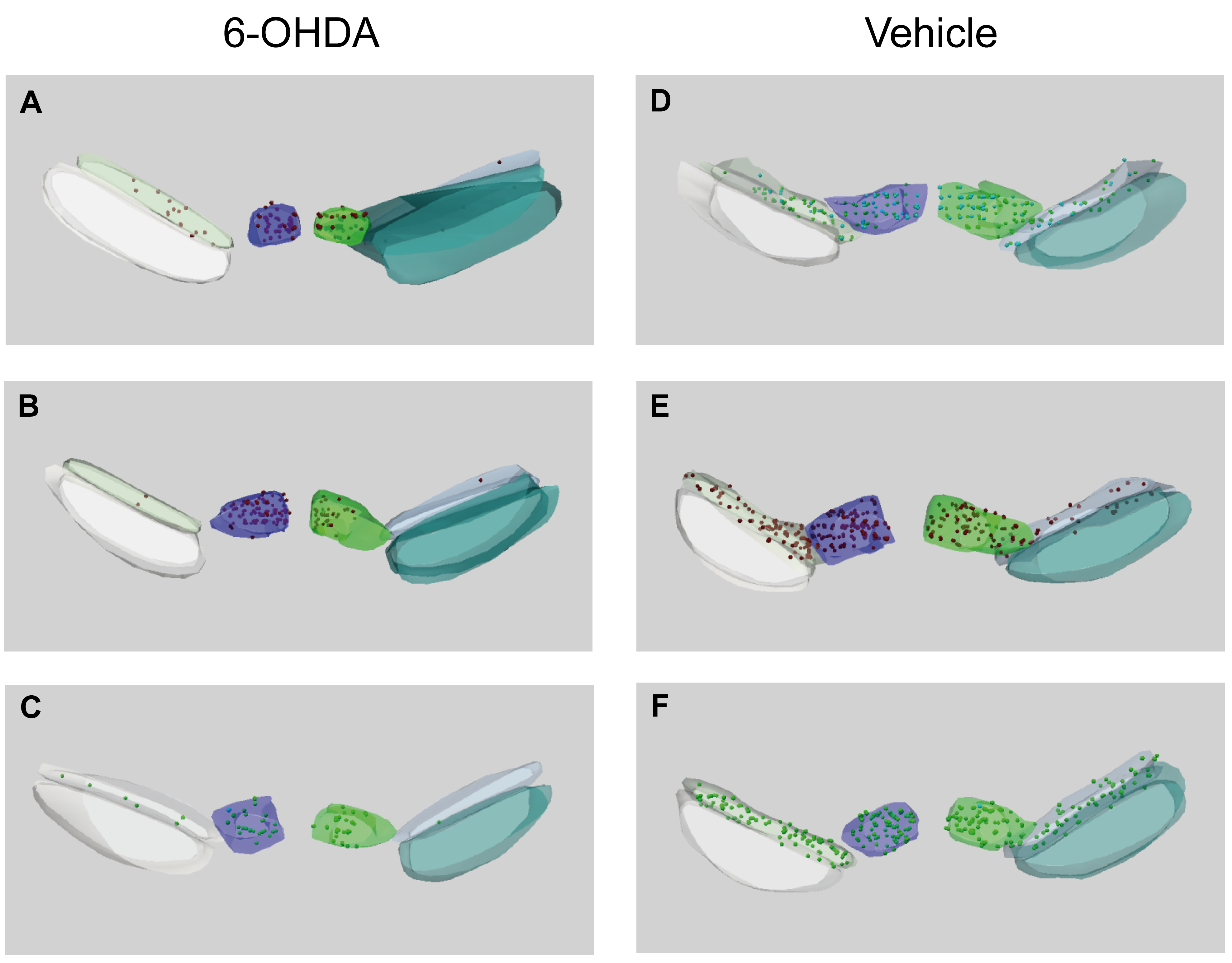

Supplement: Figure S1 — 3-D reconstructions of stereology. Panels (A-F) depict 3D-reconstructions of stereological regions of interest for all 6-OHDA (left column) or vehicle injected rats (right column) used in gene expression profiling. Different ROIs are color-coded (white: left SNr, pale green: left SNc, purple: left VTA, green: right VTA, pale blue: right SNc, cyan: right SNr). Stereological cell counting was only performed in VTA and SNc-ROIs. Spheres indicate stereologically counted cells in the SNc and VTA. 6-OHDA, 6-hydroxydopamine; ROI, region of interest; SNr, substantia nigra pars reticulata; SNc, substantia nigra pars compacta; VTA, ventral tegmental area. (TIF) [file pone.0091663.s001.tif]

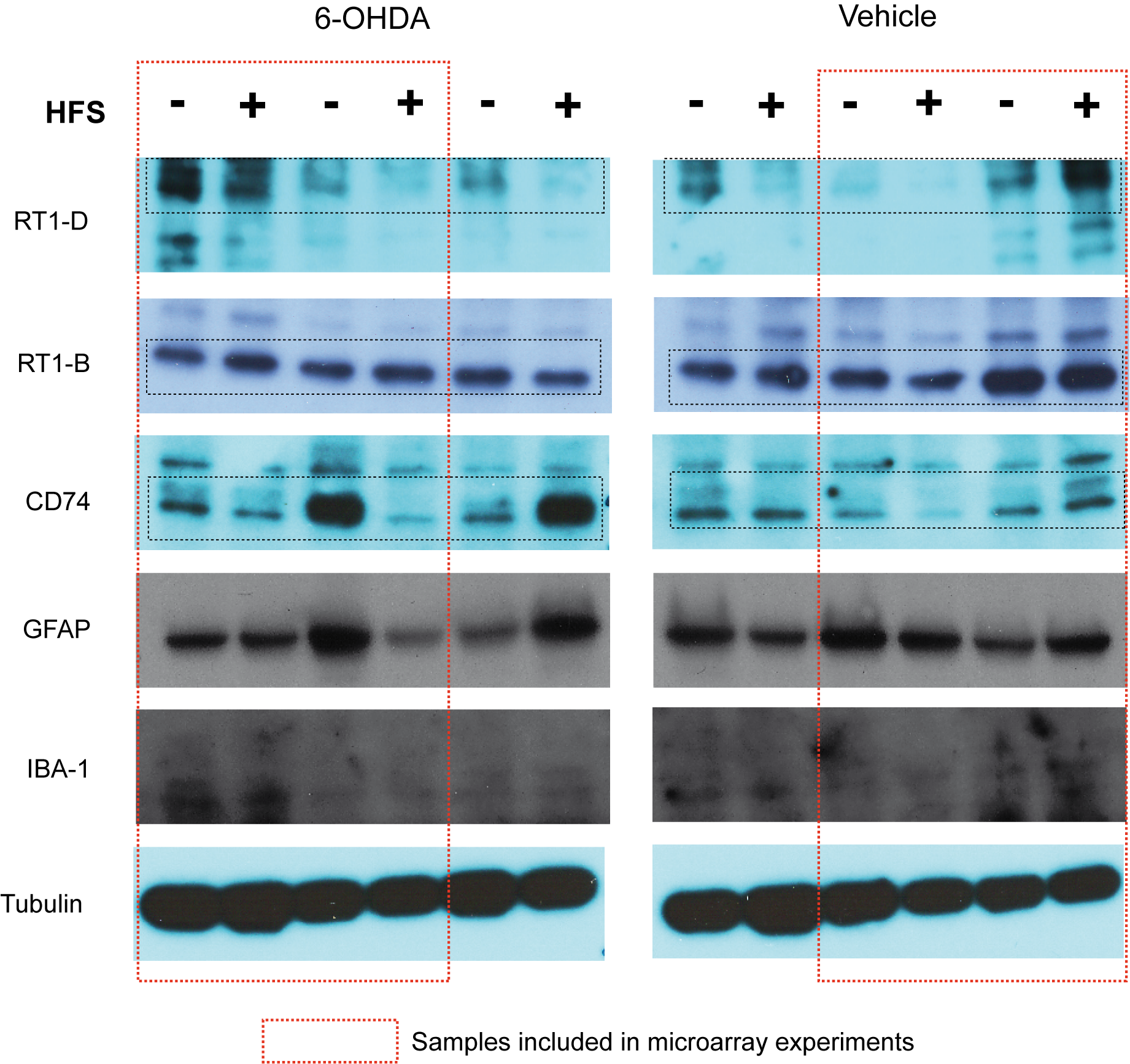

Supplement: Figure S2 — Western blot analysis. Detection of MHC class II candidate proteins (RT1-D, RT1-B, CD74), astroglial (GFAP) and microglial (Iba-1) markers by Western blot analysis. All candidates belonged to the enriched gene cluster ‘antigen processing and presentation of peptide antigen.’ The housekeeping protein tubulin served as biological control. All twelve lanes for a given antigen were blotted simultaneously and cropped later for graphical display. 6-OHDA, 6-hydroxydopamine; HFS, high-frequency stimulation; MHC, major histocompatibility complex. (TIF) [file pone.0091663.s002.tif]

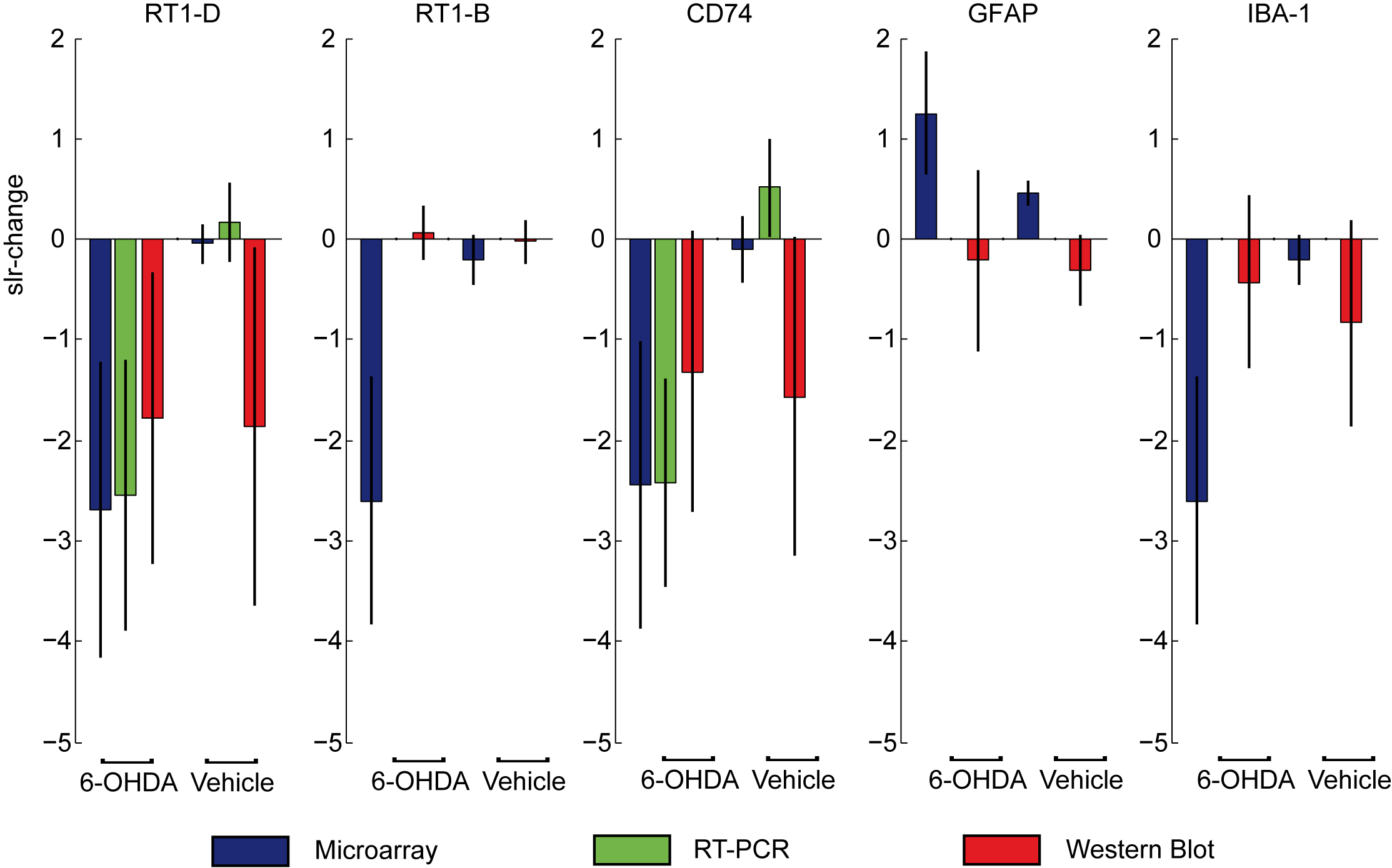

Supplement: Figure S3 — Comparison of microarray, RT-PCR and Western blot regulation changes. Quantification of signal-log ratio (slr) changes of MHC class II candidates (RT1-D, RT1-B, CD74), astroglial (GFAP) and microglial (Iba-1) markers. We utilized densitometric quantification to evaluate protein levels in Western blots. No statistical comparison was applied due to low sample size and missing data regarding RT-PCR (RT1-B, GFAP and Iba-1 were only investigated by Western blotting). (TIF) [file pone.0091663.s003.tif]
