# Supplementary material for: High-Frequency Stimulation of the Subthalamic Nucleus Counteracts Cortical Expression of Major Histocompatibility Complex Genes in a Rat Model of Parkinson’s Disease
Source: PLoS One. 2014 Mar 12;9(3):e91663. doi: 10.1371/journal.pone.0091663 (PMC3951430; doi:10.1371/journal.pone.0091663)
Supplement: Table S1 — Microarray expression values MHC class II genes. RMA-normalized microarray expression values of ‘counter-regulated’ MHC class II genes are given for single microarray experiments, respectively for both 6-OHDA and vehicle injected rats. The mean slr of the HFS-effect equals the binary logarithm of the fold change between the not stimulated and stimulated hemispheres of either 6-OHDA or vehicle injected rats. The mean slr of the lesion effect is calculated across groups between the non-stimulated hemispheres of vehicle and 6-OHDA injected rats. Counter-regulation is characterized by opposite regulation direction in the effect of lesion vs. the HFS-effect comparison. That is, STN-HFS normalizes expression values on the stimulated side of PD rats to values of controls on both stimulated and not stimulated hemispheres. RMA, robust multiarray averaging; 6-OHDA, 6-hydroxydopamine; slr, signal-log ratio; HFS, high-frequency stimulation; STN, subthalamic nucleus. (DOCX) [file pone.0091663.s004.docx]

**Table S1:**

| **Gene symbol** | **Affymetrix ID** | **6-OHDA: STN-HFS-effect** | | | | | **Vehicle: STN-HFS-effect** | | | | | **Lesion-effect** |
| --- | --- | --- | --- | --- | --- | --- | --- | --- | --- | --- | --- | --- |
|  |  | **slr** | **signal stim** | | **signal no-stim** | | **slr** | **signal stim** | | **signal no-stim** | | **slr** |
|  |  | **mean** | **MA1** | **MA2** | **MA1** | **MA2** | **mean** | **MA1** | **MA2** | **MA1** | **MA2** | **mean** |
| RT1-Db1 | 1370383_s_at | -2.7 | 49.7 | 318.5 | 1004.4 | 623.3 | - | 173.9 | 363 | 199.7 | 272.4 | 1.9 |
| RT1-Da | 1370883_at | -2.7 | 193.3 | 1157.9 | 4215.7 | 2001.5 | - | 721.5 | 1072.8 | 1007.1 | 915.3 | 1.5 |
| RT1-Bb | 1371033_at | -2.6 | 52.9 | 266.5 | 938.4 | 627.8 | - | 159.9 | 305.3 | 260 | 255.9 | 1.5 |
| Cd74 | 1367679_at | -2.4 | 402 | 2180.5 | 6785.3 | 3795.2 | - | 1769.4 | 2225.8 | 2479.7 | 1765.6 | 1.2 |
| RT1-Ba | 1381593_x_at | -2.4 | 315.1 | 1824.3 | 4493 | 3368.4 | - | 1339.3 | 2039.5 | 1874.4 | 1792.9 | 1.2 |
